# Serum soluble isoform of receptor for advanced glycation end product is a predictive biomarker for acute exacerbation of idiopathic pulmonary fibrosis: a German and Japanese cohort study

**DOI:** 10.1186/s12931-024-03014-7

**Published:** 2024-11-11

**Authors:** Erika Kitadai, Kakuhiro Yamaguchi, Shinichiro Ohshimo, Hiroshi Iwamoto, Shinjiro Sakamoto, Yasushi Horimasu, Takeshi Masuda, Taku Nakashima, Hironobu Hamada, Francesco Bonella, Josune Guzman, Ulrich Costabel, Noboru Hattori

**Affiliations:** 1 Department of Molecular and Internal Medicine, Graduate School of Biomedical and Health Sciences, Hiroshima University, 1-2-3 Kasumi, Minami-Ku, Hiroshima, 734-8551 Japan; 2 Department of Emergency and Critical Care Medicine, Graduate School of Biomedical and Health Sciences, Hiroshima University, Hiroshima, Japan; 3 Department of Physical Analysis and Therapeutic Sciences, Graduate School of Biomedical and Health Sciences, Hiroshima University, Hiroshima, Japan; 4 Center for Interstitial and Rare Lung Disease, Department of Pneumology, Ruhrlandklinik University Hospital, University of Duisburg-Essen, Essen, Germany; 5 General and Experimental Pathology, Ruhr-University, Bochum, Germany

**Keywords:** Acute exacerbation, Idiopathic pulmonary fibrosis, Rs2070600, Soluble receptor for advanced glycation end product

## Abstract

**Background:**

The receptor for advanced glycation end product (RAGE) is a transmembrane receptor accelerating a pro-inflammatory signal. RAGE signalling is promoted by decreased soluble isoform of RAGE (sRAGE), which is a decoy receptor for RAGE ligands, and RAGE SNP rs2070600 minor allele. In Caucasian and Japanese cohorts, low circulatory sRAGE levels and presence of the minor allele are associated with poor survival of idiopathic pulmonary fibrosis (IPF) and increased disease susceptibility to interstitial lung disease, respectively. However, whether sRAGE and RAGE SNP rs2070600 are associated with acute exacerbation of IPF (AE-IPF) is unclear.

**Methods:**

This retrospective cohort study evaluated the association between the onset of AE-IPF and serum sRAGE levels in 69 German and 102 Japanese patients with IPF. The association of AE-IPF with RAGE SNP rs2070600 in 51 German and 84 Japanese patients, whose DNA samples were stored, was also investigated.

**Results:**

In each cohort, the incidence of AE-IPF was significantly and reproducibly higher in the patients with sRAGE < 467.1 pg/mL. In a pooled exploratory analysis, the incidence of AE-IPF was lowest in the patients with higher sRAGE levels and rs2070600 minor allele, although no significant difference in the incidence was observed between the patients with and without the rs2070600 minor allele.

**Conclusions:**

Low sRAGE levels were associated with increased incidence of AE-IPF in two independent cohorts of different ethnicities. The combination of rs2070600 and sRAGE levels may stratify patients with IPF for the risk of AE.

**Supplementary Information:**

The online version contains supplementary material available at 10.1186/s12931-024-03014-7.

## Background

Idiopathic pulmonary fibrosis (IPF) is a progressive and irreversible fibrotic lung disease of unknown aetiology. “Acute exacerbation” is an acute complication of IPF (AE-IPF) and leads to death in approximately 40% of patients [1, 2]. However, the pathogenesis of AE-IPF remains unclear, and reproducible predictive biomarkers have not been validated.

The receptor for advanced glycation end product (RAGE) is a transmembrane receptor abundantly expressed in the healthy lung, especially in type 1 pneumocytes [3]. In RAGE knockout mice, pulmonary fibrosis develops with aging, and therefore, RAGE contributes to lung homeostasis and differentiation of pneumocytes [4]. Meanwhile, the interaction between RAGE and its ligands, including high mobility group box 1 (HMGB1) and S100 proteins, activates inflammatory signalling and exacerbates lung injury [5–7]. In patients with IPF, higher serum HMGB1 levels at diagnosis are associated with earlier onset of AE-IPF [8]. Additionally, circulatory levels of HMGB1, S100A8, and S100A9 at the onset of AE-IPF are significantly increased compared to those at diagnosis of IPF. Moreover, high levels also resulted in poor survival in patients with AE-IPF [8, 9]. Therefore, excessive RAGE signalling may be involved in the pathogenesis of AE-IPF.

RAGE signalling is aggravated by reduced soluble RAGE (sRAGE). sRAGE is a soluble isoform of RAGE without an intracellular signalling domain that circulates in the blood [10]. sRAGE is produced by enzymatic cleavage of membrane-bound RAGE and alternative splicing of the RAGE gene. sRAGE can inhibit the interaction between RAGE and ligands, thereby suppressing intracellular signal transduction and thus demonstrating its anti-inflammatory effects [11, 12]. In both Caucasian and Japanese cohorts, several reports have demonstrated that sRAGE levels in the blood are lower in patients with IPF than in healthy participants, and its low levels are associated with poor prognosis in patients with IPF [13–15]. Moreover, RAGE signalling is activated by RAGE SNP rs2070600 minor allele that increases the affinity of the ligand binding domain of RAGE [16]. In Caucasian and Japanese cohorts, the minor allele increases the disease susceptibility of interstitial lung disease including IPF [13, 14, 17]. However, whether circulatory levels of sRAGE and RAGE SNP rs2070600 are associated with the development of AE-IPF is unclear.

This study aimed to explore whether serum levels of sRAGE and RAGE SNP rs2070600 could predict AE in German and Japanese patients with IPF.

## Methods

### Patients and study design

**Fig. 1:**
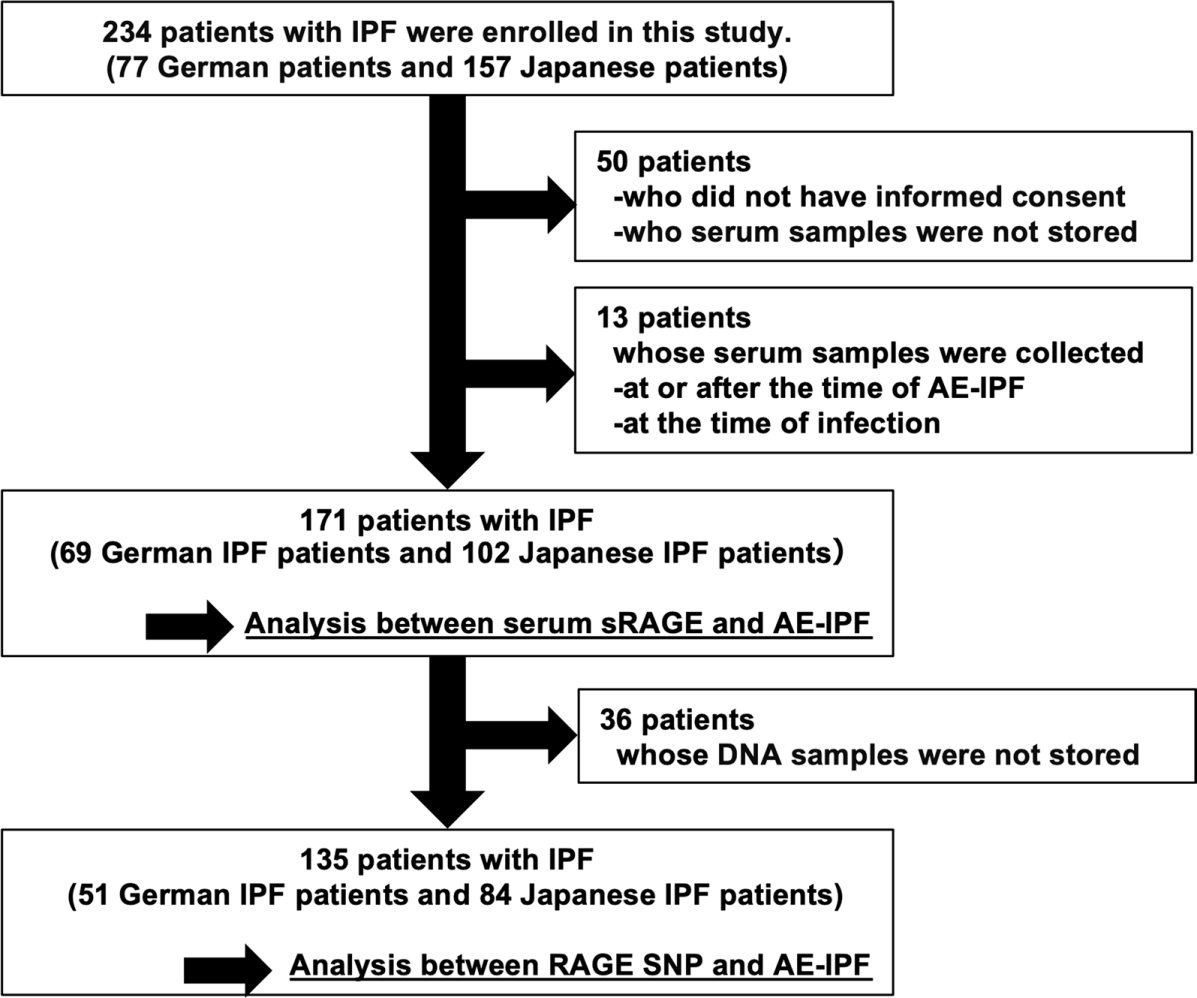
Trial profile. This study enrolled 234 patients with idiopathic pulmonary fibrosis (IPF). Sixty-three patients who did not provide informed consent or serum samples and whose serum was collected at the time of acute exacerbation of IPF (AE-IPF) or infection were excluded. Finally, 171 patients were analysed to elucidate the association between serum soluble receptor for advanced glycation end product (sRAGE) and AE-IPF. After excluding 36 patients whose DNA samples were not stored, the association between RAGE SNP and AE-IPF was analysed in 135 patients

This multicentre retrospective cohort study included 77 German and 157 Japanese patients with IPF (Fig. 1). German patients with IPF were diagnosed at Ruhrlandklinik, and Japanese patients with IPF were diagnosed at Hiroshima University Hospital. Fifty patients without informed consent or serum samples and 13 patients whose serum samples were collected during or after AE-IPF occurrence or infection were excluded. Therefore, the association between AE-IPF and serum levels of sRAGE was analysed in 69 German and 102 Japanese patients with IPF. Additionally, 36 patients did not have DNA samples. Thus, the association between AE-IPF and RAGE SNP rs2070600 was analysed in 51 German and 84 Japanese patients with IPF. IPF was diagnosed according to the criteria of the American Thoracic Society/European Respiratory Society [18]. AE-IPF was diagnosed according to an International Working Group Report [1]. This study was approved by the Ethics Committees of Ruhrlandklinik (IRB 06-3170) and Hiroshima University Hospital (IRB33 and M326) and conducted in accordance with the Declaration of Helsinki. All the patients provided written informed consent and permitted the use of their samples.

### Data collection

Patient clinical records were reviewed retrospectively. We obtained information regarding the patients’ characteristics, such as age, sex, smoking history, and pulmonary function test results. Serum and DNA samples were collected at the first visit or before treatment. Patients were censored at the date of death, at the time they were lost to follow-up, or after completing 3 years of follow-up.

### Measurement of serum sRAGE levels

Serum samples were stored at − 80 °C. Serum sRAGE levels were measured using a RAGE Quantikine ELISA kit (R&D systems, Minneapolis, Minnesota, United States).

### DNA preparation and genotype analyses of rs2070600

Peripheral whole venous blood samples were stored at − 80 °C. Genomic DNA was extracted using the phenol–chloroform extraction and ethanol precipitation methods, as previously described [19]. We used TaqMan® SNP Genotyping Assay (Thermo Fisher Scientific, Minato-ku, Tokyo, Japan) and the Applied Biosystems 7500 Fast RT-PCR System (Thermo Fisher Scientific, Minato-ku, Tokyo, Japan) for SNP typing.

### Statistical analysis

Data are shown as medians (interquartile ranges). When differences among groups were examined, the Mann–Whitney U test was used to compare continuous variables, and the chi-square test was used to compare nominal variables. The chi-square test was also used to test for deviation from Hardy–Weinberg equilibrium. The associations of AE-IPF with sRAGE, RAGE SNP rs2070600, and their combination were evaluated in a pooled cohort comprising German and Japanese patients with IPF. If significant associations were determined, reproducibility was confirmed in each cohort. The onset of AE-IPF was evaluated using the Kaplan–Meier approach and log-rank test. Cox proportional hazards analysis was performed to identify significant predictors of AE-IPF over 3 years. Receiver operating characteristic (ROC) curve analysis was performed to determine the optimal cut-off value of serum sRAGE for predicting 3-year AE-IPF. Correlations between variables were ascertained using Spearman correlation coefficients. All statistical analyses were performed using the JMP Pro 16.2.0 software (SAS Institute Japan Co., Ltd., Tokyo, Japan). Significance was set at *P* < 0.05.

## Results

### Clinical characteristics

This study included 171 patients with IPF (69 German and 102 Japanese patients). The median follow-up time was 29.9 months (12.0–44.8 months). The main characteristics of the patients are shown in Table 1. The median age was 68 years, and 139 of 171 patients (81.2%) were men. The Japanese cohort comprised more male patients with IPF than the German cohort. No significant differences in age, vital capacity (VC), and diffusing capacity for carbon monoxide (DLco) were observed between the German and Japanese patients with IPF. The German patients with IPF had significantly higher BMI and lower pack-year smoking history than the Japanese patients with IPF. Additionally, no patients had malignant tumours when serum samples were obtained, and no cancer-related deaths were recorded during the study period. No significant differences in serum sRAGE levels were observed between the German and Japanese patients with IPF [512.3 pg/mL (397.2–677.4) and 561.3 pg/mL (385.3–758.9), respectively; *P* = 0.27]. No patients were treated with corticosteroids when the serum samples were obtained.

**Table 1:** Clinical characteristics of the study participants

	All patients with IPF (n = 171)	German patients with IPF (n = 69)	Japanese patients with IPF (n = 102)	*P-*value^*^
Age, years	68 (62.0–74.0)	69 (63.0–75.0)	68 (60.8–74.0)	0.51
Sex, male/female	139/32	49/20	90/12	0.0046
BMI, kg/m^2^	24.9 (22.5–27.8)	27.7 (24.9–29.5)	23.6 (21.3–25.8)	< 0.0001
Smoking history, pack years	25.0 (0–45.0)	0.0 (0–30.0)	32.5 (15.0–48.0)	< 0.0001
VC, %predicted	71.0 (59.0–83.0)	60.0 (58.3–78.0)	72.0 (61.7–87.2)	0.10
DLco, %predicted^a^	47.2 (38.4–59.6)	44.5 (35.3–54.5)	48.4 (39.6–62.4)	0.054
sRAGE, pg/mL	524.4 (391.7–718.4)	512.3 (397.2–677.4)	561.3 (385.3–758.9)	0.27

Data are shown as medians (interquartile ranges)

*BMI* body mass index, *DLco* diffusing capacity for carbon monoxide, *sRAGE* soluble receptor for advanced glycation end product, *VC* vital capacity

*Compared between Japanese and German patients

^a^The German and Japanese cohorts had 13 and 12 missing data, respectively

### Association of AE-IPF with sRAGE

Twenty-eight patients (16.3%) developed AE-IPF over 3 years (8 German and 20 Japanese patients). Apparent triggers of AE were not identified in the 28 patients with AE-IPF. For example, bacterial infection was assessed by sputum cultures and/or urine antigen tests for Streptococcus pneumonia/Legionella pneumonia. Hence, bacteria commonly causing pneumonia were not detected at time of AE-IPF.

In the pooled cohort of German and Japanese patients with IPF, univariate Cox proportional hazards analysis showed that sRAGE (per 10 pg/mL) and VC were significantly associated with AE-IPF [hazard risk (HR) = 0.97, 95% confidential interval (CI) = 0.95–0.99, *P* = 0.0010, and HR = 0.97, 95% CI = 0.95–0.99, *P* = 0.038, respectively] (Table 2). In the multivariate analysis adjusted for VC, sRAGE (per 10 pg/mL) was significantly associated with AE-IPF (HR = 0.98, 95% CI = 0.96–0.99, *P* = 0.0067).

**Table 2:** Cox proportional hazards analysis for predicting acute exacerbation of idiopathic pulmonary fibrosis

	German + Japanese			German			Japanese		
	HR	95% CI	*P*-value	HR	95% CI	*P*-value	HR	95% CI	*P*-value
Univariate analysis									
Age, years	0.99	0.96–1.0	0.73	1.0	0.93–1.1	0.83	0.99	0.94–1.0	0.61
Sex, male	2.5	0.58–10	0.16	2.5	0.30–20	0.34	2.5	0.34–19	0.30
BMI, kg/m^2^	1.1	0.98–1.2	0.13	1.2	0.98–1.4	0.089	1.0	0.93–1.2	0.44
Smoking history, pack years	1.0	0.99–1.0	0.35	1.0	0.99–1.1	0.20	1.0	0.99–1.0	0.68
sRAGE, per 10 pg/mL	0.97	0.95–0.99	0.0010*	0.94	0.90–0.99	0.011*	0.98	0.96–0.99	0.012*
VC, %predicted	0.97	0.95–0.99	0.038*	0.93	0.88–0.98	0.0026*	0.99	0.96–1.0	0.49
Antifibrotic agent, +	1.1	0.54–2.4	0.73	NA	NA	0.66	1.1	0.44–2.6	0.88
Multivariate analysis									
sRAGE, per 10 pg/mL	0.98	0.96–0.99	0.0067*	0.95	0.91–0.99	0.011*	0.98	0.96–1.0	0.044*
VC, %predicted	0.98	0.95–1.0	0.087	0.92	0.87–0.97	0.0028*	0.99	0.96–1.0	0.65

*BMI* body mass index, *CI* confidence interval, *DLco* diffusing capacity for carbon monoxide, *HR* hazard risk, *NA* not available, *sRAGE* soluble receptor for advanced glycation end product, *VC* vital capacity

*P < 0.05, Cox proportional hazards analysis

**Fig. 2:**
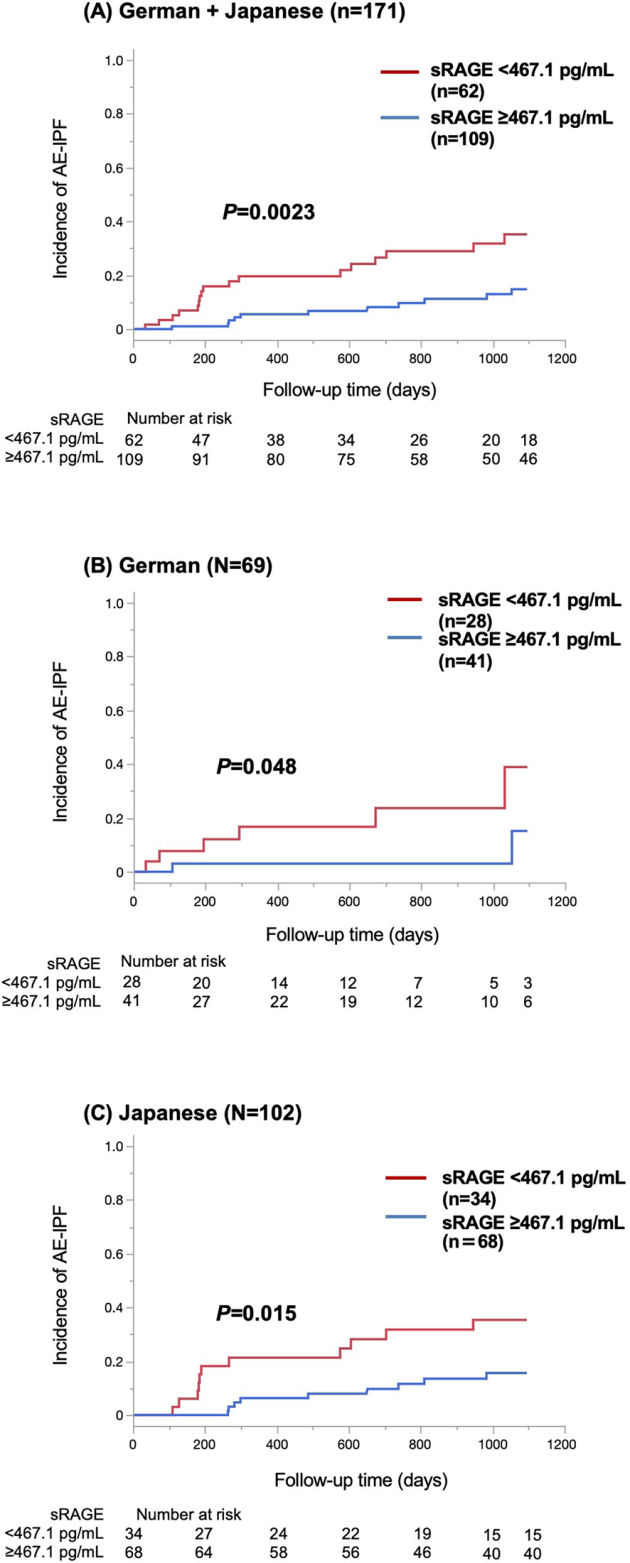
Incidence of acute exacerbation of idiopathic pulmonary fibrosis (AE-IPF) based on serum levels of serum soluble receptor for advanced glycation end product (sRAGE). The red solid line indicates the patients with low serum sRAGE levels (< 467.1 pg/mL), and the blue solid line indicates the patients with higher serum sRAGE levels (≥ 467.1 pg/mL). Patients with low serum sRAGE levels (< 467.1 pg/mL) showed a significantly higher incidence of AE-IPF than patients with higher serum sRAGE levels in the German and Japanese cohorts (**A**), German cohort (**B**), and Japanese cohort (**C**) with IPF

The significant association between low sRAGE levels and high incidence of AE-IPF was also confirmed in the German and Japanese cohorts analysed using univariate and multivariate Cox proportional hazards models (Table 2). In the Japanese cohort, the optimal cut-off level of sRAGE for predicting AE-IPF was 467.1 pg/mL (sensitivity, 60.0%; specificity, 72.0%), which was identified by ROC curve analysis (area under the curve = 0.672) (Supplemental Figure S1). Kaplan–Meier curve analysis and log-rank test revealed that sRAGE levels < 467.1 pg/mL were associated with a higher incidence of AE-IPF in the pooled cohort (*P* = 0.0023) (Fig. 2A), that was reproducibly confirmed in the German and Japanese cohorts (*P* = 0.048 and *P* = 0.015, respectively) (Fig. 2B, C).

Additionally, the association between sRAGE level and AE-IPF severity was explored in the pooled cohort. The Kaplan–Meier curve analysis and log-rank test revealed no significant difference in the prognosis between AE-IPF patients with sRAGE levels < 467.1 pg/mL (n = 17) and those without (n = 11) (Supplemental Figure S2).

### Association between baseline characteristics and serum sRAGE levels

Spearman correlation coefficients revealed that higher BMI was weakly correlated with low sRAGE levels in the pooled and Japanese cohorts (Table 3). DLco data were missing; therefore, the correlation between DLco and sRAGE levels was analysed in the pooled (n = 146), German (n = 56), and Japanese cohorts (n = 90). A positive correlation was identified between DLco and sRAGE levels in the pooled and Japanese cohorts. No factors correlated with sRAGE levels in the German cohort.

**Table 3:** Association between baseline characteristics and serum soluble receptor for advanced glycation end product levels

	German + Japanese		German		Japanese	
	ρ	*P*-value	ρ	*P*-value	ρ	*P*-value
Age	0.022	0.78	0.040	0.75	0.015	0.88
BMI	− 0.22	0.0049	0.0007	0.99	-0.27	0.0069
Pack years	0.040	0.62	0.026	0.84	-0.044	0.66
VC	0.11	0.15	0.11	0.36	0.10	0.32
DLco^a^	0.22	0.0064	0.15	0.27	0.25	0.016

*BMI* body mass index, *VC* vital capacity, *DLco* diffusing capacity for carbon monoxide, *VC* vital capacity

^a^The German and Japanese cohorts had 13 and 12 missing data, respectively

### Association of AE-IPF with RAGE SNP and the combination of sRAGE and RAGE SNP

DNA samples were preserved in 135 of 171 patients with IPF (51 of 69 German patients and 84 of 102 Japanese patients). The prevalence of the rs2070600 minor T allele was 3.9% (n = 2) and 41.7% (n = 35) in the German and Japanese cohorts, respectively (Supplemental Table S1). Although not significant, serum sRAGE levels were lower in the patients with the minor allele than those without in the pooled cohort [498.8 (300.5–680.0) and 529.5 (398.3–733.0), *P* = 0.085, respectively] (Supplemental Figure S3).

**Fig. 3:**
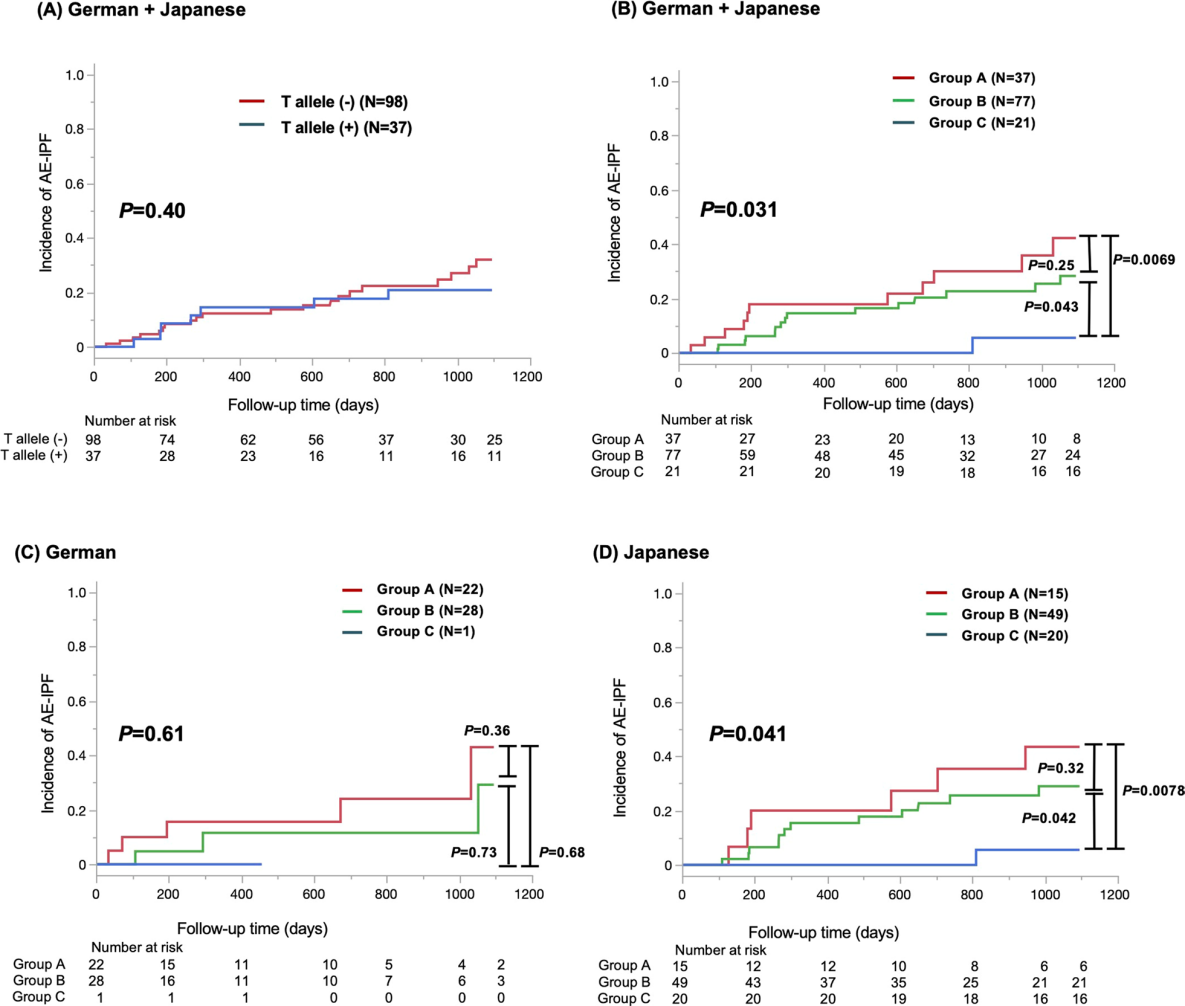
Incidence of acute exacerbation of idiopathic pulmonary fibrosis (AE-IPF) based on receptor for advanced glycation end product (RAGE) SNP rs2070600 and serum levels of serum soluble RAGE (sRAGE). In **A**, the red solid line indicates the patients without the RAGE SNP rs2070600 minor allele, and the blue solid line indicates the patients with the RAGE SNP rs2070600 minor allele. No significant difference in AE-IPF incidence was observed between the patients with and without the RAGE SNP rs2070600 minor allele. In **B**–**D**, three patient groups were defined based on sRAGE and RAGE SNP; group A (red solid line), the patients with soluble receptor for advanced glycation end product (sRAGE) levels < 467.1 pg/mL and no rs2070600 minor T allele; group B (green solid line), the patients with either the minor allele or sRAGE higher level; and group C (blue solid line), the patients with the minor allele and higher sRAGE level. In **B**, the incidence of AE-IPF in group C was the lowest among all groups. **C** and **D** presents the subset analyses of the German and Japanese cohorts, respectively

The patients were divided into three groups according to RAGE SNP and sRAGE level as follows: group A included the patients with sRAGE level < 467.1 pg/mL and no rs2070600 minor T allele; group B included those with either the minor allele or sRAGE higher level; and group C included those with the minor allele and higher sRAGE level (Supplemental Table S2). Although the Kaplan–Meier curve analysis and log rank test revealed no significant difference in the AE-IPF incidence between patients with and without the rs2070600 minor T allele (*P* = 0.40) (Fig. 3A), the incidence of AE-IPF in group C was the lowest among all the groups (Fig. 3B). The subgroup analyses in German and Japanese cohorts are presented in Fig. 3C, D, respectively. A significant difference was not observed in the German cohort owing to the low frequency of the rs2070600 minor T allele in German patients with IPF as the minor allele frequency may be generally rare in Caucasians (Fig. 3C).

## Discussion

Low serum sRAGE levels were associated with an increased incidence of AE-IPF as confirmed in two independent cohorts of German and Japanese patients with IPF. To the best of our knowledge, this is the first study reporting on a predictive blood marker for AE-IPF validated in different ethnicities. In addition, the combination of RAGE SNP rs2070600 genotype and sRAGE might help stratify patients with IPF for the risk of AE.

The association between low serum sRAGE levels and increased incidence of AE-IPF may be explained by the anti-inflammatory effect of sRAGE. sRAGE is mainly produced by enzymatic cleavage of membrane-bound RAGE and suppresses intracellular signalling by inhibiting the interactions between RAGE and ligands [11, 12]. HMGB1 is a RAGE ligand, and its higher serum level is a risk factor for developing AE-IPF [8]. In addition, HMGB1 is elevated in bronchoalveolar lavage fluid (BALF) of a rat model of lipopolysaccharide-induced lung injury, and intratracheal administration of sRAGE can attenuate lung injury [5]. Interestingly, circulatory levels of sRAGE are associated with sRAGE expression in the lung tissue and BALF [20, 21]. Based on these findings, low serum sRAGE levels might induce inflammation owing to increased RAGE signalling in the lung of patients with IPF, thereby increasing the onset of AE-IPF which is acute lung injury superimposed on pulmonary fibrosis.

Another possible explanation for the increased incidence of AE-IPF due to decreased circulatory sRAGE levels is that its low levels are correlated with decreased pulmonary function [22]. Previous reports have indicated that low plasma sRAGE levels are associated with low forced vital capacity (FVC) in a Caucasian cohort with IPF [14]. A study conducted in Spain has also revealed an association between decreased sRAGE levels and deterioration of FVC, DLco, and total lung capacity over time [15]. In line with these reports, this study also identified a significant correlation between sRAGE and DLco in the pooled and Japanese cohorts, although this correlation was not significant in the German cohort. Importantly, decreased pulmonary function parameters are risk factors for AE-IPF [1, 23–25]. Additionally, as pulmonary fibrosis progresses, RAGE expression in the lung decreases with decreasing levels of circulatory sRAGE [26]. Taken these data together, low serum sRAGE levels may reflect the progression of pulmonary fibrosis thereby being correlated with decreased pulmonary function which in turn is associated with a higher AE-IPF incidence.

This study showed that the incidence of AE-IPF could not be stratified by rs2070600 minor T allele, but the combination of the minor T allele and sRAGE may stratify the risk of AE-IPF; the incidence of AE-IPF was the lowest in the patients with the rs2070600 minor T allele and higher sRAGE levels, indicating a protective role of the minor allele only when combined with sRAGE. By contrast, the rs2070600 minor T allele, which increases the affinity of the ligand binding domain of RAGE, may promote the development of interstitial lung disease because the minor T allele is more frequent in Japanese and Caucasian patients with interstitial lung disease including IPF than in healthy participants [13, 14]. These paradoxical effects on disease susceptibility and disease progression of IPF have been also reported for several SNPs of other genes. For example, the MUC5B SNP rs35705950 minor T allele is involved in the onset of IPF, although it is involved in improving survival [27]. In addition, the FAM13A SNP rs2609255 T allele is associated with decreased disease susceptibility of IPF, although it is associated with decreased lung function and poor prognosis [28]. The protective effect of the rs2070600 minor T allele for disease progression in pulmonary diseases other than IPF has been reported; the minor allele is associated with higher FEV1/FVC in patients with chronic obstructive pulmonary disease (COPD) [29, 30]. These data support that the rs2070600 minor T allele can protect against IPF progression, although this protective effect was observed only when the rs2070600 minor T allele was combined with sRAGE.

The mechanism underlying the protective effect of the rs2070600 minor T allele on the development of AE-IPF despite the pro-inflammatory effect of the minor allele by increasing the affinity of the ligand binding domain of membrane-bound RAGE is unclear. One potential mechanism is that rs2070600 minor T allele elevates ligand binding affinity of sRAGE as well as membrane-bound RAGE. The rs2070600 minor T allele is associated with decreased circulatory sRAGE levels in various conditions including COPD and IPF [13, 14, 31]. This study also showed that serum sRAGE levels were lower in the patients with the minor T allele than in those without. Additionally, AE-IPF incidence was the lowest in the patients with the minor T allele and higher sRAGE levels. Thus, the rs2070600 minor T allele enhances the anti-inflammatory effect of sRAGE via elevating the ligand binding affinity of sRAGE, thereby increasing sRAGE consumption followed by decreasing sRAGE levels in the circulation. Therefore, in patients with the rs2070600 minor T allele and high sRAGE levels, the abundance of sRAGE with a high ability to capture RAGE ligands might highly prevent inflammation, resulting in a lower incidence and risk of AE.

This study has several limitations. First, owing to the retrospective study design, the baseline characteristics, especially sex, BMI, and smoking history, had some differences between the Japanese and German patients. Second, the frequency of the rs2070600 minor allele in the German cohort with IPF was lower than that of the Japanese cohort because the minor allele frequency is generally lower in Caucasians than in Japanese individuals [13, 14]. Third, the number of patients included in this study was relatively small, hence underpowered for subgroups analyses evaluating the predictive value of the combination of RAGE SNP and sRAGE for AE-IPF. Lastly, because no apparent triggers of AE were identified, this study consequently analysed the association between idiopathic AE and sRAGE. Further prospective studies with a larger sample size are needed to confirm the clinical utility of sRAGE for evaluating the risk of AE, including idiopathic- and triggered-AE.

## Conclusions

sRAGE can be a predictive marker for AE-IPF. Additionally, the combination of RAGE SNP rs2070600 and serum sRAGE levels may support risk stratification of AE-IPF, although further confirmative study is necessary.

## Supplementary Information

Supplementary Material 1

## Data Availability

No datasets were generated or analysed during the current study.

## Abbreviations

AE: Acute exacerbation
BMI: Body mass index
CI: Confidential interval
COPD: Chronic obstructive pulmonary disease
DLco: Diffusing capacity for carbon monoxide
FVC: Forced vital capacity
HMGB1: High mobility group box 1
HR: Hazard risk
IPF: Idiopathic pulmonary fibrosis
ND: No data
NA: Not available
RAGE: Receptor for advanced glycation end product
ROC: Receiver operating characteristic
sRAGE: Soluble receptor for advanced glycation end product
VC: Vital capacity

## References

[1] Collard HR, Ryerson CJ, Corte TJ, Jenkins G, Kondoh Y, Lederer DJ, et al. acute exacerbation of idiopathic pulmonary fibrosis. An international working group report. Am J Respir Crit Care Med. 2016;194:265–75. 10.1164/rccm.201604-0801CI. 27299520 10.1164/rccm.201604-0801CI

[2] Leuschner G, Behr J. Acute exacerbation in interstitial lung disease. Front Med. 2017;4:176. 10.3389/fmed.2017.00176. 10.3389/fmed.2017.00176 PMC5660065 29109947

[3] Demling N, Ehrhardt C, Kasper M, Laue M, Knels L, Rieber EP. Promotion of cell adherence and spreading: a novel function of RAGE, the highly selective differentiation marker of human alveolar epithelial type I cells. Cell Tissue Res. 2006;323:475–88. 10.1007/s00441-005-0069-0. 16315007 10.1007/s00441-005-0069-0

[4] Englert JM, Hanford LE, Kaminski N, Tobolewski JM, Tan RJ, Fattman CL, et al. A role for the receptor for advanced glycation end products in idiopathic pulmonary fibrosis. Am J Pathol. 2008;172:583–91. 10.2353/ajpath.2008.070569. 18245812 10.2353/ajpath.2008.070569 PMC2258251

[5] Izushi Y, Teshigawara K, Liu K, Wang D, Wake H, Takata K, et al. Soluble form of the receptor for advanced glycation end-products attenuates inflammatory pathogenesis in a rat model of lipopolysaccharide-induced lung injury. J Pharmacol Sci. 2016;130:226–34. 10.1016/j.jphs.2016.02.005. 27038888 10.1016/j.jphs.2016.02.005

[6] Blondonnet R, Audard J, Belville C, Clairefond G, Lutz J, Bouvier D, et al. RAGE inhibition reduces acute lung injury in mice. Sci Rep. 2017;7:7208. 10.1038/s41598-017-07638-2. 28775380 10.1038/s41598-017-07638-2 PMC5543147

[7] Araki K, Kinoshita R, Tomonobu N, Gohara Y, Tomida S, Takahashi Y, et al. The heterodimer S100A8/A9 is a potent therapeutic target for idiopathic pulmonary fibrosis. J Mol Med. 2021;99:131–45. 10.1007/s00109-020-02001-x. 33169236 10.1007/s00109-020-02001-x

[8] Yamaguchi K, Iwamoto H, Sakamoto S, Horimasu Y, Masuda T, Miyamoto S, et al. Serum high-mobility group box 1 is associated with the onset and severity of acute exacerbation of idiopathic pulmonary fibrosis. Respirology. 2020;25:275–80. 10.1111/resp.13634. 31270920 10.1111/resp.13634

[9] Tanaka K, Enomoto N, Hozumi H, Isayama T, Naoi H, Aono Y, et al. Serum S100A8 and S100A9 as prognostic biomarkers in acute exacerbation of idiopathic pulmonary fibrosis. Respir Investig. 2021;59:827–36. 10.1016/j.resinv.2021.05.008. 34154976 10.1016/j.resinv.2021.05.008

[10] Basta G. Receptor for advanced glycation endproducts and atherosclerosis: from basic mechanisms to clinical implications. Atherosclerosis. 2008;196:9–21. 10.1016/j.atherosclerosis.2007.07.025. 17826783 10.1016/j.atherosclerosis.2007.07.025

[11] Park L, Raman KG, Lee KJ, Lu Y, Ferran LJ Jr, Chow WS, et al. Suppression of accelerated diabetic atherosclerosis by the soluble receptor for advanced glycation endproducts. Nat Med. 1998;4:1025–31. 10.1038/2012. 9734395 10.1038/2012

[12] Yonekura H, Yamamoto Y, Sakurai S, Petrova RG, Abedin MJ, Li H, et al. Novel splice variants of the receptor for advanced glycation end-products expressed in human vascular endothelial cells and pericytes, and their putative roles in diabetes-induced vascular injury. Biochem J. 2003;370:1097–109. 10.1042/BJ20021371. 12495433 10.1042/BJ20021371 PMC1223244

[13] Yamaguchi K, Iwamoto H, Horimasu Y, Ohshimo S, Fujitaka K, Hamada H, et al. AGER gene polymorphisms and soluble receptor for advanced glycation end product in patients with idiopathic pulmonary fibrosis. Respirology. 2017;22:965–71. 10.1111/resp.12995. 28198072 10.1111/resp.12995

[14] Manichaikul A, Sun L, Borczuk AC, Onengut-Gumuscu S, Farber EA, Mathai SK, et al. Plasma soluble receptor for advanced glycation end products in idiopathic pulmonary fibrosis. Ann Am Thorac Soc. 2017;14:628–35. 10.1513/AnnalsATS.201606-485OC. 28248552 10.1513/AnnalsATS.201606-485OC PMC5427736

[15] Machahua C, Montes-Worboys A, Planas-Cerezales L, Buendia-Flores R, Molina-Molina M, Vicens-Zygmunt V. Serum AGE/RAGEs as potential biomarker in idiopathic pulmonary fibrosis. Respir Res. 2018;19:215. 10.1186/s12931-018-0924-7. 30409203 10.1186/s12931-018-0924-7 PMC6225674

[16] Hofmann MA, Drury S, Hudson BI, Gleason MR, Qu W, Lu Y, et al. RAGE and arthritis: the G82S polymorphism amplifies the inflammatory response. Genes Immun. 2002;3:123–35. 10.1038/sj.gene.6363861. 12070776 10.1038/sj.gene.6363861

[17] Kinjo T, Kitaguchi Y, Droma Y, Yasuo M, Wada Y, Ueno F, et al. The Gly82Ser mutation in AGER contributes to pathogenesis of pulmonary fibrosis in combined pulmonary fibrosis and emphysema (CPFE) in Japanese patients. Sci Rep. 2020;10:12811. 10.1038/s41598-020-69184-8. 32732977 10.1038/s41598-020-69184-8 PMC7393115

[18] Raghu G, Remy-Jardin M, Myers JL, Richeldi L, Ryerson CJ, Lederer DJ, et al. Diagnosis of idiopathic pulmonary fibrosis. An official ATS/ERS/JRS/ALAT clinical practice guideline. Am J Respir Crit Care Med. 2018;198:e44–68. 10.1164/rccm.201807-1255ST. 30168753 10.1164/rccm.201807-1255ST

[19] Hiyama E, Yokohama T, Hiyama K, Yamakido M, Santo T, Kodama T, et al. Alteration of telomeric repeat length in adult and childhood solid neoplasias. Int J Oncol. 1995;6:13–6. 10.3892/ijo.6.1.13. 21556494 10.3892/ijo.6.1.13

[20] Nakao S, Yamaguchi K, Iwamoto H, Kagimoto A, Mimae T, Tsutani Y, et al. Role of soluble receptor for advanced glycation end products in postoperative fibrotic lung injury. Ann Thorac Surg. 2022;113:1617–23. 10.1016/j.athoracsur.2021.05.059. 34139190 10.1016/j.athoracsur.2021.05.059

[21] Yamaguchi K, Iwamoto H, Mazur W, Miura S, Sakamoto S, Horimasu Y, et al. Reduced endogenous secretory RAGE in blood and bronchoalveolar lavage fluid is associated with poor prognosis in idiopathic pulmonary fibrosis. Respir Res. 2020;21:145. 10.1186/s12931-020-01410-3. 32527263 10.1186/s12931-020-01410-3 PMC7291663

[22] Keefe J, Yao C, Hwang SJ, Courchesne P, Lee GY, Dupuis J, et al. An integrative genomic strategy identifies sRAGE as a causal and protective biomarker of lung function. Chest. 2022;161:76–84. 10.1016/j.chest.2021.06.053. 34237330 10.1016/j.chest.2021.06.053 PMC8783029

[23] Song JW, Hong SB, Lim CM, Koh Y, Kim DS. Acute exacerbation of idiopathic pulmonary fibrosis: incidence, risk factors and outcome. Eur Respir J. 2011;37:356–63. 10.1183/09031936.00159709. 20595144 10.1183/09031936.00159709

[24] Mura M, Porretta MA, Bargagli E, Sergiacomi G, Zompatori M, Sverzellati N, et al. Predicting survival in newly diagnosed idiopathic pulmonary fibrosis: a 3-year prospective study. Eur Respir J. 2012;40:101–9. 10.1183/09031936.00106011. 22241745 10.1183/09031936.00106011

[25] Costabel U, Inoue Y, Richeldi L, Collard HR, Tschoepe I, Stowasser S, et al. Efficacy of nintedanib in idiopathic pulmonary fibrosis across prespecified subgroups in INPULSIS. Am J Respir Crit Care Med. 2016;193:178–85. 10.1164/rccm.201503-0562OC. 26393389 10.1164/rccm.201503-0562OC

[26] Ohlmeier S, Mazur W, Salmenkivi K, Myllärniemi M, Bergmann U, Kinnula VL. Proteomic studies on receptor for advanced glycation end product variants in idiopathic pulmonary fibrosis and chronic obstructive pulmonary disease. Proteom Clin Appl. 2010;4:97–105. 10.1002/prca.200900128. (**Epub 2010 Jan 7**). 10.1002/prca.200900128 21137019

[27] Peljto AL, Zhang Y, Fingerlin TE, Ma SF, Garcia JG, Richards TJ, et al. Association between the MUC5B promoter polymorphism and survival in patients with idiopathic pulmonary fibrosis. JAMA. 2013;309:2232–9. 10.1001/jama.2013.5827. 23695349 10.1001/jama.2013.5827 PMC4545271

[28] Hirano C, Ohshimo S, Horimasu Y, Iwamoto H, Fujitaka K, Hamada H, et al. FAM13A polymorphism as a prognostic factor in patients with idiopathic pulmonary fibrosis. Respir Med. 2017;123:105–9. 10.1016/j.rmed.2016.12.007. 28137485 10.1016/j.rmed.2016.12.007

[29] Repapi E, Sayers I, Wain LV, Burton PR, Johnson T, Obeidat M, et al. Genome-wide association study identifies five loci associated with lung function. Nat Genet. 2010;42:36–44. 10.1038/ng.501. 20010834 10.1038/ng.501 PMC2862965

[30] Hancock DB, Eijgelsheim M, Wilk JB, Gharib SA, Loehr LR, Marciante KD, et al. Meta-analyses of genome-wide association studies identify multiple loci associated with pulmonary function. Nat Genet. 2010;42:45–52. 10.1038/ng.500. 20010835 10.1038/ng.500 PMC2832852

[31] Cheng DT, Kim DK, Cockayne DA, Belousov A, Bitter H, Cho MH, et al. Systemic soluble receptor for advanced glycation endproducts is a biomarker of emphysema and associated with AGER genetic variants in patients with chronic obstructive pulmonary disease. Am J Respir Crit Care Med. 2013;188:948–57. 10.1164/rccm.201302-0247OC. 23947473 10.1164/rccm.201302-0247OC

