# Supplementary material for: Serum soluble isoform of receptor for advanced glycation end product is a predictive biomarker for acute exacerbation of idiopathic pulmonary fibrosis: a German and Japanese cohort study
Source: Respir Res. 2024 Nov 11;25:405. doi: 10.1186/s12931-024-03014-7 (PMC11552171; doi:10.1186/s12931-024-03014-7)
Supplement: Supplementary file 1 — Supplementary Material 1 [file 12931_2024_3014_MOESM1_ESM.docx]

**Additional files**

**Supplemental Figure S1.** ROC curve for predicting AE-IPF

**AUC 0.672**

**1-Specificity**

**Sensitivity**

**1.00**

**0.80**

**0.60**

**0.40**

**0.20**

**0.00**

**0.00**

**0.20**

**0.40**

**0.60**

**0.80**

**1.00**

ROC curve analysis identified 467.1 pg/mL as the optimal cut-off level of sRAGE for predicting AE-IPF (AUC = 0.672). The cut-off level had a sensitivity and specificity of 60.0% and 72.0%, respectively.

AE, acute exacerbation; AUC, area under the curve; IPF, idiopathic pulmonary fibrosis; ROC, receiver operating characteristic; sRAGE, soluble receptor for advanced glycation end product.

**Supplemental Figure S2.** Association between serum sRAGE level and prognosis of patients with AE-IPF

**
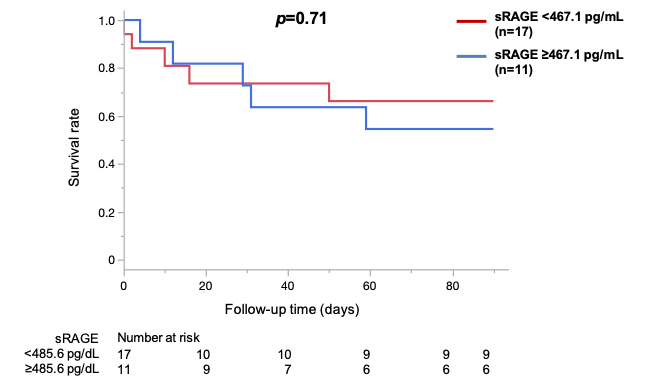
**

Kaplan–Meier curve analysis and log-rank test revealed no significant difference in the prognosis between AE-IPF patients with sRAGE levels < 467.1 pg/mL (n=17) and those without (n=11) (log-rank test *P*=0.71).

*P* values were determined by using log-rank test.

AE, acute exacerbation; IPF, idiopathic pulmonary fibrosis; sRAGE, soluble receptor for advanced glycation end product.

**Supplemental Figure S3.** Association between serum sRAGE level and SNP (rs2070600 minor T allele)


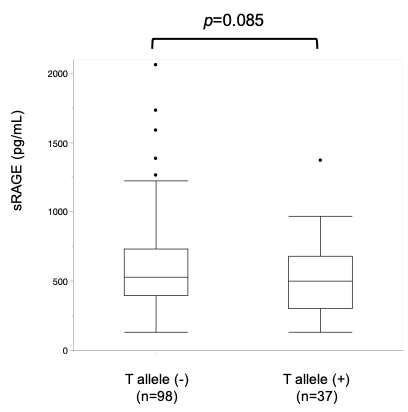


Serum sRAGE levels were lower in the patients with the minor allele than those without it in the pooled cohort [498.8 (300.5–680.0) and 529.5 (398.3–733.0), *P*=0.085, respectively].

sRAGE, soluble receptor for advanced glycation end product.

**Supplemental Table S1.** Genotype distributions and allele frequency of rs2070600 in German and Japanese patients with IPF

| Genotype | German | Japanese |
| --- | --- | --- |
| CC, n (%) | 49 (96.1) | 49 (58.3) |
| CT, n (%) | 2 (3.9) | 34 (40.5) |
| TT, n (%) | 0 (0) | 1 (1.2) |
| HWE p-value | 1.00 | 0.291 |
| MAF (T), % | 2.0 | 21.4 |

HWE, Hardy–Weinberg equilibrium; MAF, minor allele frequencies.

**Supplemental Table S2.** Clinical characteristics of the study participants with DNA samples.

|  | ALL (n=135) |  | Rs2070600 T allele | |  | Combination of rs2070600 and sRAGE | | |
| --- | --- | --- | --- | --- | --- | --- | --- | --- |
|  |  |  | Absent  (n=98) | Present  (n=37) |  | Group A* (n=37) | Group B* (n=77) | Group C* (n=21) |
| Age, years | 68  (63–74) |  | 69  (63–75.3) | 67  (58.5–73.5) |  | 69  (63.5–73) | 68  (61.5–76) | 68  (59.5–74) |
| Sex, male/female | 109/26 |  | 77/21 | 32/5 |  | 30/7 | 61/16 | 18/3 |
| BMI, kg/m^2^ | 24.9  (22.5–28.4) |  | 25.0  (22.7–28.4) | 23.5  (22.1–27.9) |  | 26.1  (24.5–28.3) | 24.9  (21.8–29.2) | 23.0  (20.2–24.9) |
| Smoking history, pack years | 27  (0–44.5) |  | 20  (0–40.0) | 37.5  (25.5–59.0) |  | 20  (0–31.9) | 28.5  (0–45.0) | 30  (25.0–52.5) |
| VC, %predicted | 70.8  (58.6–84.5) |  | 72.0  (60.8–85) | 65.3  (55.4–81.2) |  | 65  (55–78.3) | 73  (65.0–88.1) | 65.6  (53.1–80.6) |
| DLco, %predicted** | 47.7  (38.5–59.6) |  | 47.5  (38.7–61.3) | 47.9  (38.3–59.3) |  | 45  (35–61.1) | 48.1  (39.2–57.5) | 49.2  (41.8–64.4) |

Data are shown as medians (interquartile ranges).

BMI, body mass index; DLco, diffusing capacity for carbon monoxide; ND, no data; sRAGE, soluble receptor for advance glycation end product; VC, vital capacity.

*Group A, patients with sRAGE level < 467.1 pg/mL and no rs2070600 minor T allele; group B, patients with either the minor allele or higher sRAGE level; and group C, patients with the minor allele and higher sRAGE level.

**Data were missing in 20 patients.
